# NCAPG2 could be an immunological and prognostic biomarker: From pan-cancer analysis to pancreatic cancer validation

**DOI:** 10.3389/fimmu.2023.1097403

**Published:** 2023-01-27

**Authors:** Qi Wang, Zhangzuo Li, Shujing Zhou, Zhengrui Li, Xufeng Huang, Yiwei He, Yuhan Zhang, Xiaoxian Zhao, Yidan Tang, Min Xu

**Affiliations:** ^1^ Department of Gastroenterology, Affiliated Hospital of Jiangsu University, Jiangsu University, Zhenjiang, China; ^2^ Department of Cell Biology, School of Medicine, Jiangsu University, Zhenjiang, China; ^3^ Faculty of Medicine, University of Debrecen, Debrecen, Hungary; ^4^ Department of Oral and Maxillofacial-Head and Neck Oncology, Shanghai Ninth People’s Hospital, Shanghai Jiao Tong University School of Medicine, College of Stomatology, Shanghai Jiao Tong University, Shanghai, China; ^5^ National Center for Stomatology and National Clinical Research Center for Oral Diseases, Shanghai JiaoTong University, Shanghai, China; ^6^ Shanghai Key Laboratory of Stomatology, Shanghai JiaoTong University, Shanghai, China; ^7^ Faculty of Dentistry, University of Debrecen, Debrecen, Hungary; ^8^ Key Laboratory of Cardiovascular and Cerebrovascular Medicine, Nanjing Medical University, Nanjing, China

**Keywords:** NCAPG2, pan-cancer, immune infiltration, biomarker, pancreatic cancer, immunity

## Abstract

More recently, NCAPG2 has emerged as an intrinsically essential participant of the condensin II complex involved in the process of chromosome cohesion and stabilization in mitosis, and its position in particular tumours is now being highlighted. Simultaneously, the genetic properties of NCAPG2 hint that it might have enormous potential to interpret the malignant progression of tumors in a broader perspective, that is, in pan-cancer. Yet, at present, this recognition remains merely superficial and there is a lack of more detailed studies to explore the underlying pathogenesis. To meet this need, the current study was undertaken to comprehensively elucidate the potential functions of NCAPG2 in pan-cancer, based on a combination of existing databases like TCGA and GTEx. NCAPG2 was identified to be overexpressed in almost every tumor and to exhibit significant prognostic and diagnostic efficacy. Furthermore, the correlation between NCAPG2 and selected immune features, namely immune cell infiltration, immune checkpoint genes, TMB, MSI, etc. also indicates that NCAPG2 could potentially be applied in guidance of immunotherapy. Subsequently, in pancreatic cancer, this study further clarified the utility of NCAPG2 that downregulation of its expression could result in reduced proliferation, invasion and metastasis of pancreatic cancer cells, among such phenotypical changes, the epithelial-mesenchymal transition disruption could be at least one of the possible mechanisms raising or enhancing tumorigenesis. Taken above, NCAPG2, as a member of pan-oncogenes, would serve as a biomarker and potential therapeutic target for a range of malignancies, sharing new insights into precision medicine.

## 1 Introduction

With cancer being a globally prominent public health issue, long considered the second leading cause of global death after cardio-vascular disorders, it is receiving a battle from cancer for dominance of this top position (1). Even though overall mortality rates have effectively been controlled as a result of research into its pathogenesis, diagnosis and treatment approaches since the turn of the century, insights into its biological characteristics have not met the requirements of translational medicine, nor have such efforts led to the formation of a closed loop for effective long-term management of the cancer, with the result that the annual incidence rate persists (2–4). Currently, passion for the fight against cancer is high in the medical community, yet along with it comes incessant concern (3, 5). Mutations of the genome alongside epigenetic variations render the tumor indifferent to inhibitory responses in the process of proliferation, and consequently acquiring features like uncontrolled metabolism and immune escape (6, 7). According to the hypothesis that cancer is a genetic disorder, the accumulation of such mutations generates a cascade in cell multiplication, uncontrolled differentiation, etc. (8) Above described processes arise from the omission of chromatin organization for the regulation of the cell cycle along with the maintenance of cytogenetic homeostasis, where properly and stably structured mitotic chromosomes is deemed required (9).

Homologous condensin and stabilization maintenance was confirmed to be induced by the condensin complex, a conserved multisubunit protein complex capable of regulating mitotic interphase gene expression (10). Remarkably, NCAPG2, an inaccessible component of the condensin II complex, could serve as a mediator in the microtubule-attachment process, namely by recruiting PLK1 so as to accelerate the mitotic process and thereby assist in the completion of mitosis (11, 12). Such findings undoubtedly imply that NCAPG2 might play a key role in maintaining the homeostatic survival of carcinomas and could be a promising target for cancer therapy owing to its remarkable ability to control intermitotic chromatin condensation (9, 10, 13). Evidence confirms this to be the case, as first identified by Shiheido et al. who showed that the elotinib analogue Q15 resulted in abnormal cell division of cells and apoptosis *via* chromosomal dissociation by impacting NCAPG2 expression (14). It has been established that NCAPG2 overexpression could potentially alter the signaling pathways, like STAT3 and NF-κB, or other oncogenes to promote proliferation and invasion of the malignancy cells in various cancers including liver cancer, melanoma, lung cancer and glioma (12, 15–17). Additionally, Jiang et al. shed further light on the potential of NCAPG2 to enhance the stemness of lung adenocarcinoma by acting on MYC-related pathways to generate resistance to Erlotinib (18).

Yet, although NCAPG2 has been previously documented in the before mentioned cancer types, studies in the context of the entire cancer spectrum, namely the pan-cancer, are currently absent. Utilizing public databases, pan-cancer analysis enables the most efficient understanding of the molecular mechanisms and predictive value of a gene in tumor biology, thereby maximizing the options for clinical diagnosis and treatment (19–22). Accordingly, in the present study, we leveraged resources from databases such as TCGA and GTEx to conduct a pan-cancer analysis for NCAPG2 and to investigate its implications in cancer prognosis. Moreover, in order to deeply analyze the association between NCAPG2 and tumor immunity, the study evaluated the relevance of its expression to immune checkpoint-related genes and immune cell infiltration scores. Based on this, the relationship between tumor mutational burden (TMB), microsatellite instability (MSI) and tumor stemness score was also investigated to better dissect its value in the direction of tumor immunotherapy, providing new insights into precision medicine.

## 2 Methods

### 2.1 Acquisition of pan-cancer expression data

The collated data of 10,534 TCGA pan-cancer samples and the 15,776 data integrated with the GTEx (https://commonfund.nih.gov/GTEx/) resource were downloaded from the UCSC Xena database and then log2(x+0.001) transformation was performed, which includes mRNA sequencing data for NCAPG2 in 34 different types of tumor tissues and 31 normal tissues, as well as relevant clinical data (survival status, clinical and pathological stage) (23). Cancer with less than 3 samples in a single species were discarded in this process (23–26). The R package “limma”, “ggplot2” and “ggpubr” were used to compare the expression of NCAPG2. Acquisition and graphic presentation of NCAPG2 expression at different clinical stages in specific cancer types were performed by the UALCAN platform (http://ualcan.path.uab.edu/analysis-prot.html) (27).

### 2.2 Prognostic assessment in pan-cancer

Incomplete survival information and survival status samples from pan-cancer expression data and clinical information were removed to obtain collated, high-quality prognostic expression data. A univariate COX regression model was developed using the R package “survival” and the Kaplan-Meier curve was plotted for each cancer type. The prognostic value of NCAPG2 in different cancer types was assessed by 4 types of clinical outcomes: overall survival (OS), disease-specific survival (DSS), disease-free interval (DFS), and progression-free interval (PFS). Prognostic indicators were assessed on criteria covering hazard ratios (HR), 95% confidence intervals and p-values indicated when p<0.05 was considered statistically significant.

### 2.3 Exploring the relationship between NCAPG2 and clinical features

The correlation analysis between the clinical stage and the expression of NCAPG2 was done by the R packages “limma” and “ggpubr”. The R packages “pROC” and “ggplot2” were then used to calculate the ROC-AUC values of NCAPG2 at the pan-cancer level. In principle, AUC values above 0.8 are considered to be of high reliability.

### 2.4 Correlation analysis of immune traits

Firstly, NCAPG2 and 60 immune checkpoint pathway genes and 41 chemokines, 18 chemokine receptors and 21 MHC-related immune pathway signatures genes were collected from a standardized pan-cancer gene expression dataset (28). The relationship between the expression levels was reasoned by the R package “limma” and the correlation coefficient was determined by the Pearson statistic approach. Then, the StromalScore, ImmuneScore, and ESTIMATE scores were calculated for 10,180 tumor samples from 44 tumor types by the R package “ESTIMATE” (29). The Spearman correlation between genes and immune infiltration scores in individual tumours was then calculated by applying the corr.test function of the R package psych, which in turn identified statistically significantly associated immune infiltration scores. Lastly, immune cell infiltration results for 33 cancers were downloaded from the TIMER 2.0 database (http://timer.cistrome.org) for comparison (30). Combining the collated gene expression data, the infiltration scores of B cell, T cell, Neutrophil, Macrophage, DC, and other immune cells were measured for every patient in each tumor using the “Timer”, “deconvo_epic”, and “deconvo_quantiseq” algorithms through the R package “IOBR” (31–33). The results were visualized by the R packages “reshape2” and “RColorBreyer”.

### 2.5 Association of NCAPG2 with genomic heterogeneity and stemness

It is now widely accepted that genomic heterogeneity of tumours closely impacts the response to treatment with immune checkpoint inhibitors (ICIS) and patient prognosis, including TMB, MSI, purity and other indicators (34, 35). MSI scores and tumor purity data for an individual tumor were obtained from the previous study by Thorsson V et al. The collated gene expression data were integrated with the MSI scores and purity data from the pan-cancer samples and Spearman correlation analysis was performed (28). Processed TCGA samples of diverse cancers were downloaded from the GDC online database (https://portal.gdc.cancer.gov/) and further characterized for correlation of TMB with gene expression (36). The TMB score was mainly implemented with the assistance of the TMB function in the R package “maftools”. The tumor stemness score relies mainly on the OCLR algorithm from Tarthiane-M et al, which is obtained by calculating the DNAss index of diverse malignancies by methylation characteristics (37). Then, the stemness scores and gene expression data of the samples were integrated to derive the Pearson correlation between the two and visualized.

### 2.6 Cell culture

Four human pancreatic cancer (PC) cell lines (PaTu8988, PANC1, MIAPaCa-2, BXPC-3) identified by STR karyotype analysis were contributed by CAS (Shang-hai Institutes for Biological Sciences). All cells were cultured in DMEM (HyClone) supplemented with 10% Fetal Bovine Serum (FBS; Gibco, The USA). Cells were cultured in a 5% CO2 incubator set at a constant temperature of 37°C.

### 2.7 Real-time PCR

Total RNA was isolated and extracted using the Manufacturers recommended RNAiso Plus (Takara, Dalian, China) and RevertAid First Strand cDNA Synthesis Kit (Thermo Fisher Scientific, Waltham, MA, USA) was used to reverse transcribe RNA into cDNA. The qPCR assay was conducted on a CFX96 real-time PCR instrument using GAPDH as an internal parameter. As follows were the primers used in the procedure: GAPDH-F:GGTGAAGGTCGGTGTGAACG, GAPDH-R:CTCGCTCCTGGAAGATGGTG; NCAPG2-F: AACCAAGCCAACATCTCCAG, NCAPG2-R: AAATCCCACCCTTTCCCTATT. Comparative expression results were calculated using the 2-ΔΔCt methodology.

### 2.8 Transfection

The vectors, sh-NC, sh-NCAPG2 were transfected into MIAPaCa-2 as well as PANC1 cells by Liposome 2000 transfection agent (Invitgen, USA) according to the manufacturer’s protocol. Sequences of shRNAs were: shRNA-1: AAAGCTGATTCACGTTATT; shRNA-2: CACGTTATTCGTCATTGCTTA; shRNA-3:AACGTCAAAGGCAGATCTGGA; shRNA-4: TGGTTATTAATGCAGGTAA; sh-NC: TTCTCCGAACGTGTCACGT.

### 2.9 Western blotting

Treated with cell lysis buffer at 100°C for a total of 10 min with cells that had been previously rinsed with cold PBS to extract total protein. Prepared proteins were added to a 10% SDS/PAGE grid system and equal amounts of proteins were electrolysed at constant pressure (200V) and then transferred to a PVDF membrane. After sealing the membrane with 5% skimmed milk powder for 1h, incubate overnight at 4°C with primary antibodies, followed by 1.5h incubation with secondary antibodies. Following incubation, the PVDF membrane strips were washed and visualized, then relative protein abundance was assessed using Image Lab analysis software and ImageJ.

### 2.10 Cell proliferation and colony formation assay

The shRNA-transfected PANC1 and MIAPaCa-2 cells were fully digested, inoculated into 96-well culture plates at 2 x 10^3 cells per well and incubated at 37°C. The absorptivity was measured every day using the CCK-8 kit, for a total of seven days of incubation. The above procedures were carried out at 450 nm on the advice of the reagent supplier and four secondary wells were retained for the experimental sessions. A total of 750 transfected control or NCAPG2 shRNA-transfected PANC1, MIAPaCa-2 cells were positioned on a fresh six-well plate and cultured for 2 weeks as described in the detail culture procedure above, during which times the medium was changed every 4 days. Formed colonies were fixed in Methanol and stained with Crystalline Violet (Sigma-Aldrich) after meeting a scheduled period, then counted manually and photographed.

### 2.11 Transwell, invasion and wound healing assay

Migration and invasion assay of cells were tested as described below using 24-well transwell chambers. PANC1, MIAPaCa-2 transfected and control cell suspension containing 50,000 cells (100 μL) were inoculated individually in transwell chambers with or without matrigel and conducted transwell assay as well as invasion assay respectively, and the culture medium containing 10% FBS was loaded into the lower chamber. The cells were incubated in the incubator for 24 h and then taken out of fixation and stained with 0.05% crystal violet dye for 30 min before being placed under the microscope for imaging. Evenly transfected PC cells were inoculated in 6-well plates and cultured until no empty space remained at the bottom. Then scraped the cells with the tip of a sterile 10-μL pipette tip, which was then counted as 0 hour, and marked it. After washing off the cell debris with PBS, the cells were incubated for 48 h. The migration of the cells was observed at 0 h, 24 h and 48 h and the migration rate was calculated.

### 2.12 Statistical analysis

A comparison of gene expression differences was performed using the Wilcoxon Rank Sum Test and the Kruskal-Wallis Test. Spearman or Pearson correlation analysis was employed to calculate the correlation between the two groups. Kaplan-Meier method and Cox regression analysis were used to compare survival characteristics. Chi-square tests and Fisher’s exact test were applied to analyze clinical characteristics. The above statistical analysis and visualization was performed with R software and GraphPad Prism 9. P < 0.05 (*), P < 0.01 (**) and P < 0.001 (***), were considered significant.

## 3 Result

### 3.1 Expression of NCAPG2 in pan-cancer

**Figure 1:**
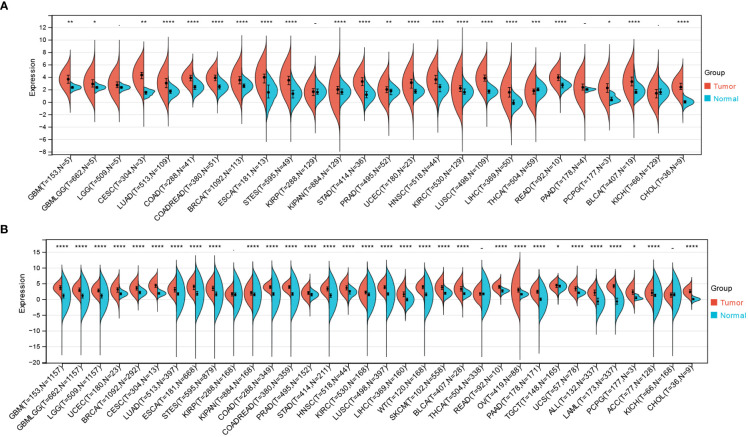
Differential expression of NCAPG2 in pan-cancer. **(A)** Analysis of NCAPG2 expression in matched tumor tissues and normal tissues using data from the TCGA database. **(B)** Matched analysis comparing NCAPG2 expression differential in TCGA database and GTEx database. Ns, p≥0.05; *p<0.05; **p<0.01; ***p<0.001; ****p<0.0001.

By combining and digging into the resources of TCGA, GTEx databases, we obtained the expression levels of NCAPG2 under a pan-cancer perspective, indicating that NCAPG2 was statistically over-expressed in 22 common malignancies (**Figure 1A**), including GBM, CESC, LUAD and others. Further expanded the normal sample size revealed that NCAPG2 was aberrantly highly expressed in practically the entire spectrum of cancers except KIRP, KICH, THCA (**Figure 1B**), among which, the most significant of these cancers tend to be LGG (p=1.1e-186), LUAD (p=6.8e-79), LUSC (p=1.5e-124), and BRCA (p=2.8e-94).

### 3.2 Survival analysis

**Figure 2:**
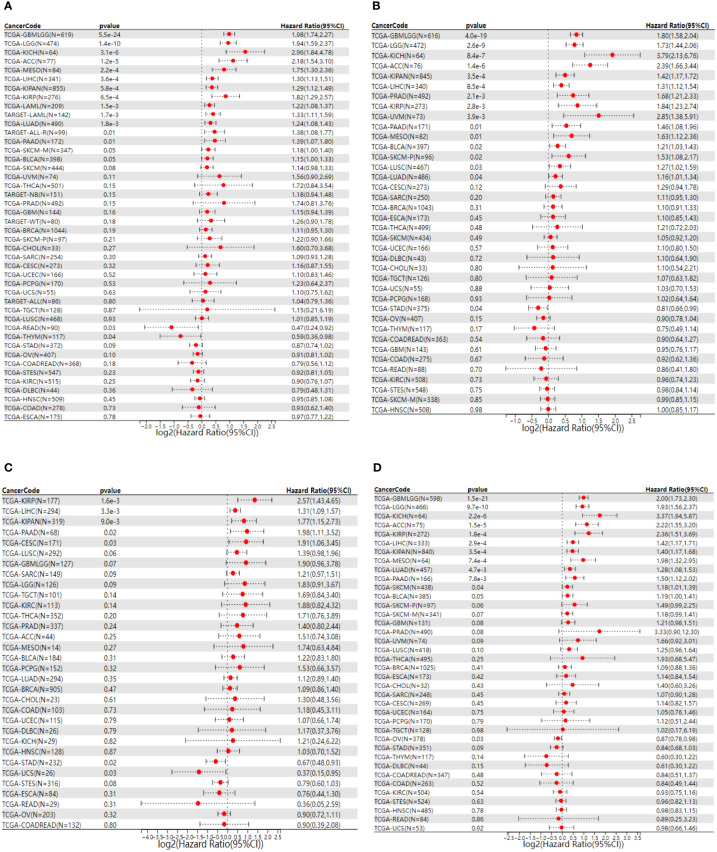
Univariate COX regression analysis of NCAPG2 with OS, PFS, DFS and DSS in pan-cancer. **(A)** Correlation between NCAPG2 expression and OS; **(B)**, DSS; **(C)**, DFS; **(D)**, PFS. OS, overall survival; DSS, disease-specific survival; DFS, disease-free survival; PFS, progression-free survival.

Survival analysis of NCAPG2 in four domains including OS, DSS, PFS and DFS reveals its prognostic value in pan-cancer. Cox regression model analysis demonstrated that high expression of NCAPG2 was a risk factor for OS in patients with the according 13 tumor types (**Figure 2A**), including LGG, LAML, LUAD, KIRP, KIPAN, LIHC, MESO, PAAD, ACC, TCGA-KICH. Further research revealed that NCAPG2 expression was also markedly associated with DSS in an array of carcinoma categories, such as LG, LUAD, KIRP, KIPAN, LIHC, SKCM, BLCA, MESO, PAAD, ACC, KICH (**Figure 2B**). In addition, high expression of NCAPG2 was furthermore suggestive of lower DFS in CESC, KIRP, KIPA, LIHC, PAAD and others (**Figure 2C**). Univariate cox regression model was further developed to correlate NCAPG2 expression with PFS in diverse cancers, which shows that NCAPG2 expression were associated with poor prognosis in 15 tumor types, including GBMLGG, LGG, LUAD, KIRP, KIPAN, PRAD, LUS, LIHC, BLCA, MESO, UVM, PAAD, ACC and KICH (**Figure 2D**). The above relationships between NCAPG2 and OS, DSS, DFS and PFS were then further elucidated by Kaplan-Meier (KM) survival analysis curves, with the overall trend being approximately the similar to that of the univariate cox regression. In KIRP, KIPAN, LIHC, LGG, LAML, LUAD, MESO, PAAD, ACC and KICH, OS showed a decreasing trend with higher NCAPG2 expression (**Figure S1**), as did the KM curves of DSS in LGG, LUAD, KIRP, KIPAN, LIHC, SKCM, BLCA, MESO, PAAD, ACC and KICH, while in READ and THYM it was accompanied by a higher OS (**Figure S2**). K-M survival analysis likewise displayed that high NCAPG expression correlated with DFS in CESC, KIRP, LIHC, and PAAD, whereas STAD, UCS manifested a higher DFS (**Figure S3**). NCAPG2 was even more significantly linked to PFS in pan-cancer, with low PFS seen in LGG, LUAD, KIRP, PRAD, LUSC, LIHC, BLCA, MESO, UVM, PAAD, ACC and KICH (**Figure S4**).

### 3.3 Relationship of NCAPG2 to clinical and pathological stage

**Figure 3:**
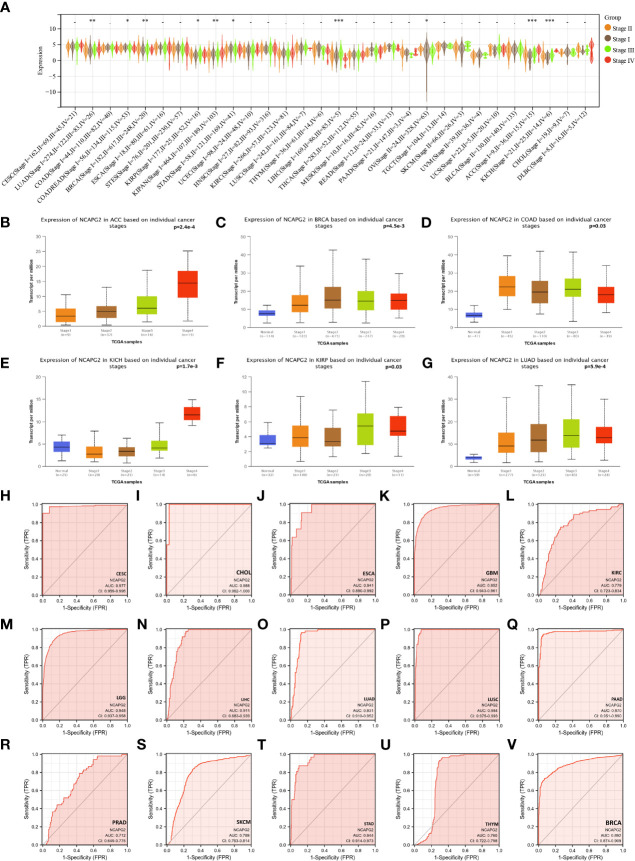
The relationship between NCAPG2 and stages and its diagnostic value. **(A)** Expression levels of NCAPG2 at distinct stages in pan-cancer; **(B–G)** NCAPG2 expression at diverse tumor stages in ACC, BRCA, COAD, KICH, KIRP, LUAD; **(H–V)** ROC curves for NCAPG2 in pan-cancer. *p<0.05; **p<0.01; ***p<0.001.

Upon further assessment of NCAPG2 expression levels at different clinical and pathological stages from a pan-cancer angle, significant difference was observed in 11 tumors (**Figure 3A**). The higher-ranking cancer types are shown in (**Figures 3B–G**). Notably, although no statistical significance was observed in tumors like LGG, MESO, CHOL, PAAD, an overall trend could be noticed that NCAPG2 is inclined to manifest a specific elevation in advanced tumors, which highlights the prognostic value of NCAPG2 to some extent. Subsequently, the ROC curves for each cancer type were plotted under the pan-cancer level, and the results showed that NCAPG2 had high accuracy (AUC>0.8) for the diagnosis of 18 cancers. Specifically: BLCA (AUC=0.882), CESC (AUC=0.977), CHOL (AUC=0.988), COAD (AUC=0.957), ESCA (AUC=0.941), GBM (AUC=0.952), HNSC (AUC=0.874), LGG (AUC=0.948), LIHC (AUC=0.911), LUAD (AUC=0.931), LUSC (AUC=0.984), OV (AUC=0.877), PAAD (AUC=0.970), READ (AUC=0.970), STAD (AUC=0.944), some of the results are given in (**Figures 3H–V**).

### 3.4 NCAPG2 is tightly related to immune infiltration and immune checkpoints

**Figure 4:**
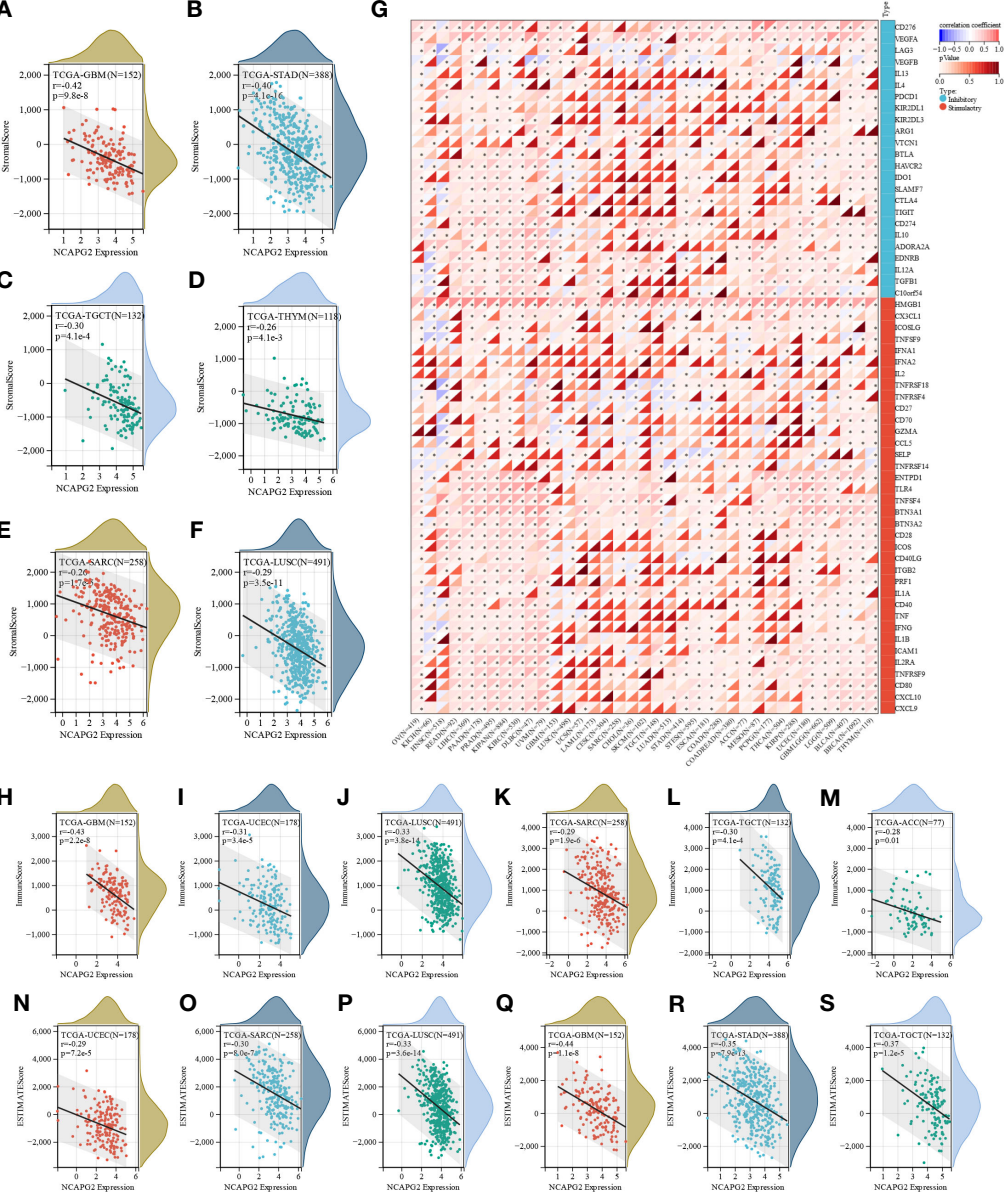
The correlation between NCAPG2 expression and immune infiltration and immune checkpoints. **(A–F)** Correlation of NCAPG2 expression with StromalScore; **(G)** Correlation of NCAPG2 expression with immune checkpoint-related genes; **(H–M)** Correlation of NCAPG2 expression with ImmuneScore; **(N–S)** Correlation of NCAPG2 expression with EstimateScore.

Next, for the purpose of exploring the significance of NCAPG2 on the tumor microenvironment (TME) by investigating the relationship between NCAPG2 and the level of immune infiltration in pan-cancer. The correlation between NCAPG2 and three immune scores was evaluated separately. (**Figures 4A–F**) presents the highest correlations of the different scores for these cancers. According to the StromalScore, NCAPG2 expression in GBM, STAD, TGCT, THYM, SARC and LUSC was significantly negative correlated with immune infiltration. ImmuneScore revealed that the expression of NCAPG2 in GBM, LUSC, SARC, TGCT, ACC and UCEC was negatively correlated with immune infiltration (**Figures 4H–M**). This trend was also suggested by the EstimateScore, where the level of immune infiltration in UCEC, SARC, LUSC, STAD, GBM, TGCT, ACC, and TGCT decreased with the expression of NCAPG2 (**Figures 4N–S**). Although the three scores differed numerically, the overall trend expressed consistency, suggesting that NCAPG2 significantly regulates TIME somewhat in the aforementioned malignancies. We compared the potential correlation of NCAPG2 expression with 60 immune checkpoint pathway genes in pan-cancer. The results are illustrated in (**Figure 4G**), where NCAPG2 demonstrated remarkable correlations with the currently listed immunosuppressive/immunostimulatory genes in pan-cancer, with the majority of positively correlated immune checkpoint pathway genes. While NCAPG2 was predominantly positively correlated with the immune checkpoint genes involved in the comparison in PAAD as well as in PRAD, negatively correlations predominated in HNSC as well as in KICH, although there was also a strong correlation. Further comparison of the results revealed that HMGB1 and NCAPG2 were all significantly positively related in pan-cancer, highlighting the necessity to further investigate the mechanisms involved. Unlike HMGB1, IFNA1, IFNA2, IL-2, KIR2DL3, IL-13 and NCAPG2 demonstrated an intuitive correlation in only a few cancer types.

### 3.5 NCAPG2 interacts strongly with immune cells

**Figure 5:**
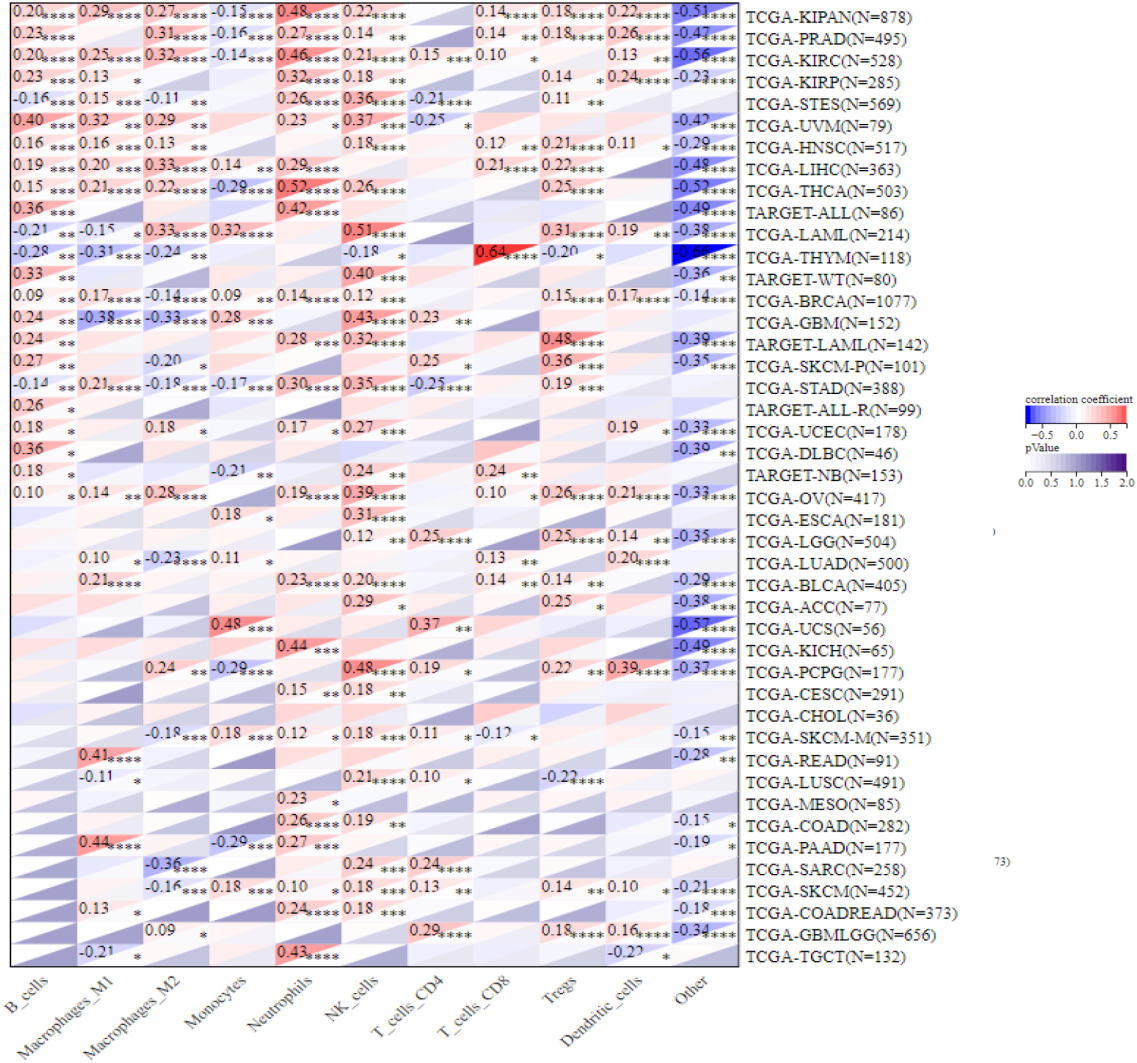
Correlation of NCAPG2 with the level of immune infiltrating cells. NCAPG2 was closely related to the immune infiltration level in cancers analyzed via QUANTISEQ algorithms. *p<0.05; **p<0.01; ***p<0.001; ****p<0.0001.

We further integrated QUANTISEQ, EPIC, and TIMER, three well-established algorithms designed to assess cross-tumor immune scoring, to assess the correlation between NCAPG2 expression and immune cell levels, building on the analysis of immune infiltration. The results demonstrated that NCAPG2 expression significantly contributed to the level of immune cell infiltration in the majority of cancer types, with macrophages, natural killer cells and neutrophils being the three immune cell types most closely related to NCAPG2 expression (**Figure 5**; **Figure S5A, B**). Among them, all three algorithms revealed that NCAPG2 was significantly and positively correlated with T cells content in THYM. Furthermore, integrating the findings of the three algorithms, we found that NCAPG2 was statistically significant negatively correlated with the infiltration level of immune cells in LUSC, TGCT, SKCM, ESCA, BLCA and other malignancies, driving the formation of an immunosuppressive microenvironment in part. Upon further comparison of the results of the three algorithms, we found that, intriguingly, despite the possible differences in the results of the three algorithms, NCAPG2 was closely correlated with diverse immune cells in LIHC, KIRC, KIPAN, PRAD, PAAD, LGG and others, showing either a strong positive or significant negative correlation, which indicates potential for further study of immune cell infiltration in the microenvironment of these cancers (**Figure 5**; **Figure S5A, B**). Notably, although NCAPG2 significantly contributed to the infiltration of immune cells overall, this relationship was not prominent in CHOL, COAD and other cancer types (**Figure 5**).

### 3.6 NCAPG2 correlates strongly with immunomodulatory genes

**Figure 6:**
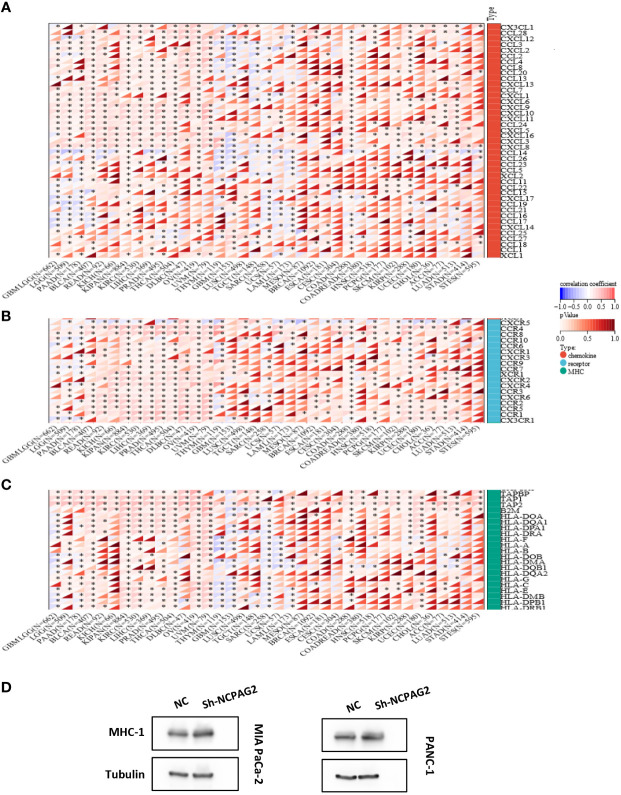
Correlation of NCAPG2 with the immunomodulatory genes. **(A)** NCAPG2 was closely related to the chemokine genes; **(B)** Correlation of NCAPG2 expression with chemokine receptor genes; **(C)** Correlation of NCAPG2 expression with MHC genes. *p<0.05; **p<0.01; ***p<0.001; **(D)** Expression differences of MHC-1 after NCAPG2 knockdown.

Furthermore, comparing NCAPG2 with immunomodulatory-related genes, including histocompatibility complex (MHC), chemokine and chemokine receptor genes, it was found that the three gene families were strongly correlated with NCAPG2 expression levels in most tumor types (**Figures 6A–C**). Among the malignancies such as LGG, PAAD, BLCA, READ, KICH, KIRC, LIHC, PRAD, THCA, OV, UVM, this correlation was more pronounced in all three gene families, with an overall positive correlation. Of these, LGG, BLCA, KIPAN, OV and UVM were at the top of the list, with NCAPG2 expression levels showing positive correlations with almost all MHC and chemokine receptor genes in these five malignancies. Despite this correlation being evident in pan-cancer, as described above, it was not significant in MESO, BRCA, ESCA, CESC, STAD and STES tumors (**Figures 6A–C**). It is worth mentioning that NCAPG2 expression levels in STES correlated with only a few immunomodulatory genes, such as CCR10 in chemokine receptor genes, B2M and TAP1 in MHC genes (**Figures 6A, B**). Besides, in addition to the vast majority of cancer species showing positive correlations, statistically significant negative correlations could be observed in THYM, GBM, LUSC, TGCT, UCS, LAML and other malignancies. Within this, the most significant is SARC, for example, the expression level of NCAPG2 in SARC is almost entirely negatively correlated with MHC genes, and only TAPBP is positively correlated (**Figure 6C**). Similar negative correlations could be observed to some extent in GBM as well as THYM, but in general not as significant as in SARC (**Figures 6A–C**). In PAAD, the expression correlation between several MHC-related genes involved in this study and NCAPG2 was predominantly in a negative direction, mainly for HLA family genes. For this reason, we further evidenced this relationship by WB experiment. We compared the changes in the expression level of MHC-1 after NCAPG2 knockdown and the results displayed that the expressed amount of MHC-1 was elevated (**Figures 6D**).

### 3.7 Prediction of treatment response to ICIS by NCAPG2

**Figure 7:**
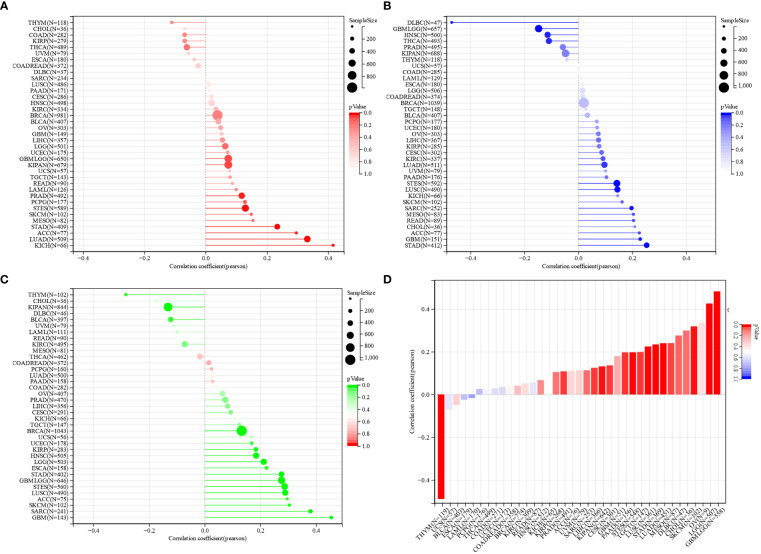
Prediction of treatment response to ICIS by NCAPG2 **(A)** Correlation analysis of the association between NCAPG2 expression and TMB, **(B)** MSI, **(C)** tumour purity and **(D)** stemness score.

As TMB and MSI seem to be the key factors in determining whether to undertake an immune checkpoint therapy or not, we have investigated and compared the correlation between NCAPG2 expression and TMB as well as MSI in a pan-cancer. Whether TMB or MSI, the expression of NCAPG2 in a pan-cancer was mostly positively correlated with its expression level. According to KICH, LUAD, ACC, STAD, MESO, TMB scores were most tightly correlated with NCAPG2 (**Figure 7A**), while NCAPG2 in patients with STAD, GBM, ACC, CHOL, READ, MESO was more closely correlated with MSI (**Figure 7B**). Although the negative correlation between NCAPG2 and TMB was not significant, DLBC, HNSC and THCA presented a much more significant negative correlation. Tumor purity is also thought to impinge on the efficacy of ICI treatment, and NCAPG2 displayed a significant correlation with purity, which was more prominent in GBM, SARC, SKCM, ACC, LUSC, STAD and others, with some similarity to TMB and MSI (**Figure 7C**). Besides, the stemness score is thought to be associated with the generation of drug resistance during the therapy of malignant tumors and the continuous proliferation of tumor cells, for this reason, we also evaluated the Pearson correlation between the expression of NCAPG2 and the stemness scores of distinct tumors. The results are presented in (**Figure 7D**), where distinct tumor stemness scores were significantly correlated with NCAPG2 in 15 tumors, with a significant positive correlation in 14 of these. Among the most significantly correlated cancer species were GBMLGG (R=0.48), LGG (R=0.43), SKCM (R=0.32), and LUAD (R=0.24) (**Figure 7D**). Of note, in THYM, this relationship displayed a significant negative correlation (R = -0.49).

### 3.8 NCAPG2 serves as a biomarker for pancreatic cancer

**Figure 8:**
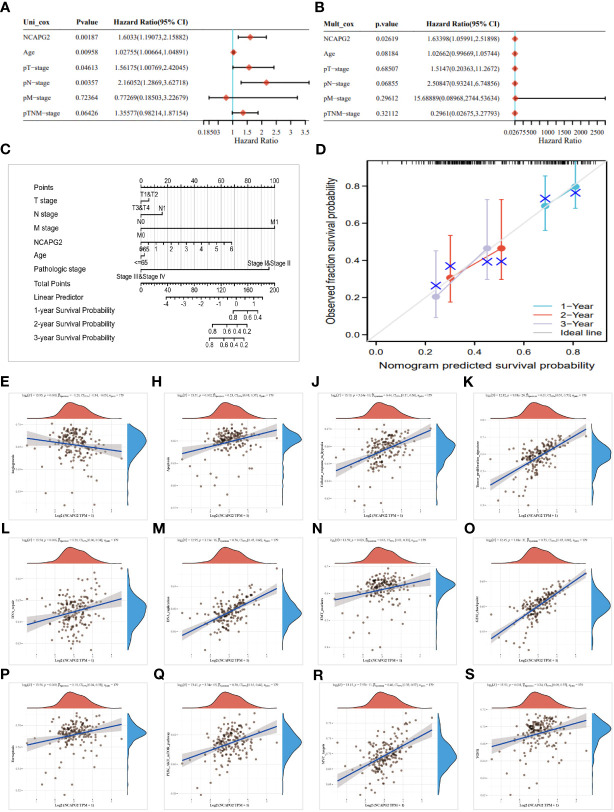
Analysis of the clinical relevance of NCAPG2 in pancreatic cancer. **(A, B)** Prognostic significance of NCAPG2 in pancreatic cancer by univariate and multifactorial COX analysis. **(C)** Nomogram based on NCAPG2 expression and pathological staging. **(D)** Correction analysis diagram of the nomogram. **(E–S)** GSVA pathway correlation analysis.

The above studies identified that NCAPG2 was significantly over-expressed in PC and that survival analysis demonstrated that NCAPG2 expression levels were strongly correlated with OS (p=0.01), DSS (p=7.8e-3), PFS (p=0.01), and DFS (p=0.02) in PC patients. Such correlations were also significant in the correlation analysis of TMB, MSI and stemness scores. For this reason, we next explored the possible biological functions and mechanisms of NCAPG2 in the context of PC. Initially we further evaluated the prognostic value of NCAPG2 in PC using univariate and multivariate COX regression models, both of which resulted in NCAPG2 being considered as an independent prognostic standard (**Figures 8A, B**). Furthermore, we constructed a nomography based on NCAPG2 expression and pathological stage to better assist in assessing patient prognosis in clinical practice (**Figure 8C**). Moreover, the calibration curves were used to assess the accuracy of the current model in assessing the prognosis of PC patients at 1, 2 and 3 years and the results were shown in (**Figure 8D**), which indicate favorable assessment performance. To better reveal the potential mechanisms by which NCAPG2 impacts on patient prognosis. We calculated the correlation between gene expression and pathway scores and found that NCAPG2 expression was significantly associated with angiogenesis, apoptosis, cellular response to hypoxia, DNA repair, tumor proliferation, DNA replication, glycolysis, EMT, ferroptosis, G2M checkpoint in PC (**Figures 8E–P**). Also based on the GSVA results, we hypothesized that the above-mentioned behavior towards influencing malignant growth of PC might be achieved by affecting signaling communication through the PI3K-AKT-mTOR pathway, MYC pathway, TGF-β pathway, etc. Therefore, we conducted an experimental study on the possibility of the above mechanisms (**Figures 8Q–S**).

### 3.9 Role of NCAPG2 on proliferation, migration and invasion of PC cells

**Figure 9:**
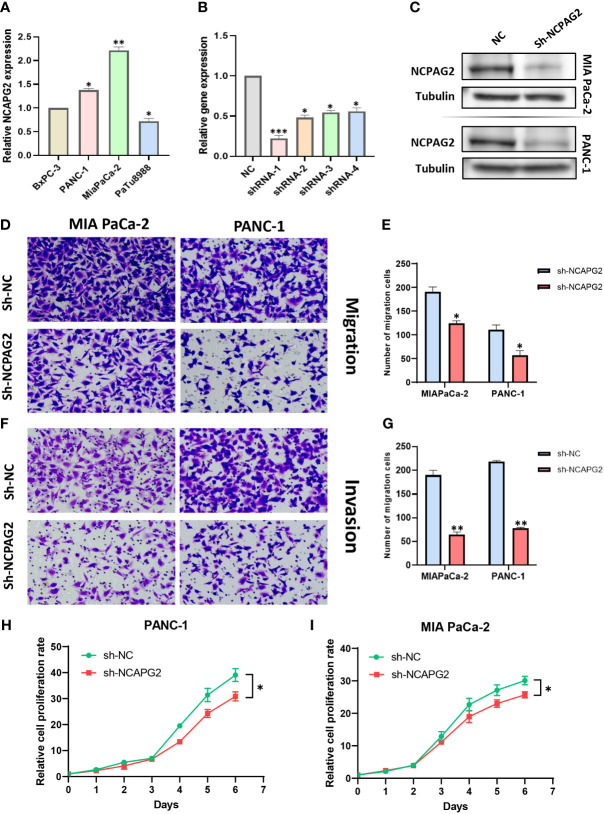
The effect of sh-NCAPG2 on the proliferation, invasion and metastasis of pancreatic cancer cells. **(A)** Relative mRNA expression of NCAPG2 in PC cell lines **(B)** and Weston blotting **(C)** determine the efficiency of sh-NCAPG2 in PC cells. **(D, F)** Migration and invasion of cells were detected employing the tranwell assay. **(E, G)** Histogram showing the number of migrating and invading cells,the error bars of the histograms indicate the SD of the three measurements. **(H, I)** CCK8 assay to detect the proliferative capacity of pancreatic cancer cells. *p< 0.05; **p<0.01; ***p<0.001.

**Figure 10:**
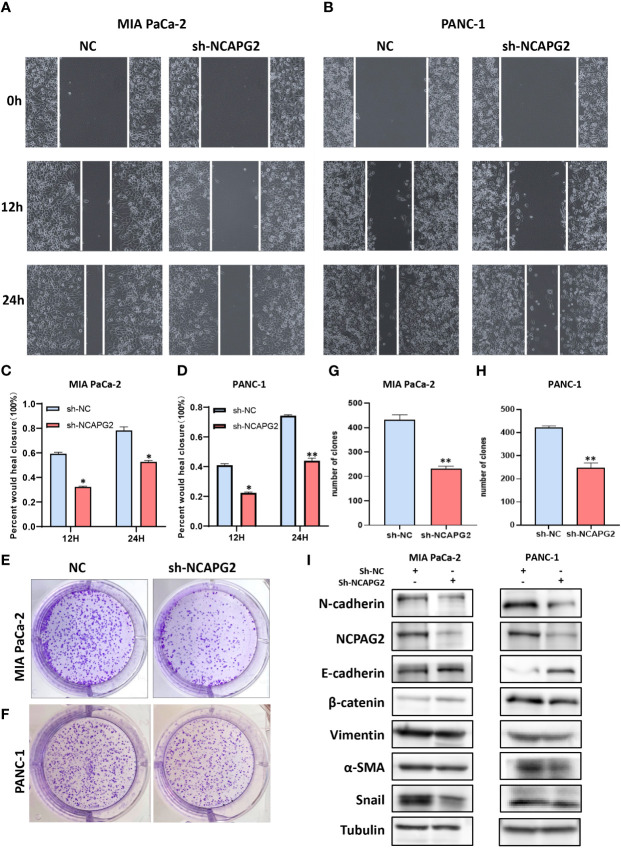
Functional effects of NCAPG2 on PC cells and their mechanisms. **(A, B)** Cell scratch assay detects migration ability; (C,D) Histogram shows the relative area of wound healing. **(E, F)** Colony formation assay was performed to compare the effect of NCAPG2 on proliferation. **(G, H)** Histograms shows the number of colony formation. **(I)** Western blotting detected the effect of sh-NCAPG2 on EMT markers. *p< 0.05; **p<0.01.

As aforementioned results have demonstrated the fact that the expression levels of NCAPG2 in tumor samples and healthy controls are not only distinctly different but also tightly associated with all the four types of survivals (OS, DSS, DFS, PFS) in pancreatic cancer, we decided to conduct further experiments to explore its general functionality in pancreatic cancer. Given the relatively high expression of NCAPG2 in both PANC1 and MIAPaCa-2 cells, we opted for these two cell lines to be transfected and carried out subsequent experiments (**Figure 9A**). The three NCAPG2 shRNA knockdown vectors were transfected into PANC1 and MIAPaCa-2 cells. The transfection efficiency was then verified by RT-PCR and Western blotting, respectively. The results are presented in (**Figures 9B, C**), shRNA-1 had the highest transfection efficiency and the knockdown efficiency reached about 80%, so was picked as the plasmid vector for further research. The ability of NCAPG2 to effectively affect the proliferation of PC cells was confirmed by CCK8 assay (**Figures 9H, I**) and colony formation assay (**Figures 10E–H**), which showed that NCAPG2 knockdown markedly reduced the proliferation ability of PC cells in PANC1 and MIAPaCa-2 compared to the control group, which was also supported by the colony formation assay. Following this, the transwell and invasion assay was applied to test the migration and invasion ability of selected cells, and the transwell assay proved that the migration ability of tumor cells was significantly reduced when NCAPG2 was down-regulated in PANC1, which was also evident in MIAPaCa-2 (**Figures 9D, E**). Further, in the invasion assay, it was observed that NCAPG2 knockdown resulted in a statistically significant reduction in the amount of tumor cells travelling through the Matrigel (**Figures 9F, G**). These findings suggest that NCAPG2 could significantly affect the proliferation, migration and invasion of pancreatic cancer cells. Not only that, we also performed a wound healing assay and the results were the same as the above findings, with the NCAPG2 knockdown group having a remarkably lower healing capacity than the control group (**Figures 10A–D**).

### 3.10 NCAPG2 boosts EMT in pancreatic cancer cells

Based on the above findings, when NCAPG2 expression was increased, a significant reduction in proliferation, migration and invasion was observed in the targeted pancreatic cancer cell lines. Furthermore, in relation to the GSVA results, it could be speculated that the mechanisms involved might be related to tumor angiogenesis or DNA repair, as well as the mechanisms involved in influencing the EMT process could also be included in our enrichment results. Epithelial-mesenchymal transition (EMT) is deemed broadly involved in the malignant progression of PC. To this end, we confirmed the correlation between EMT markers and NCAPG2 expression by employing Western blotting. As shown in the results, the expression of N-cadherin, β-catenin, Vimentin, α-SMA, and Snail was significantly downregulated in both PC cells when NCAPG2 expression was suppressed, whereas E-cadherin levels were upregulated, suggesting that downregulation of NCAPG2 could contain the EMT phenomenon in tumor cells (**Figure 10I**).

## 4 Discussion

The most intuitive and pervasive properties of malignancies could be found in uncontrolled proliferation, which extends to evasion of growth inhibitors, induction of angiogenesis, resistance to cell death, distant-metastasis and so forth hallmarks (6, 38, 39). These traits or mechanisms strongly depend on a range of alterations in the tumor cell genome, including karyotypic instability plus amplification and deletion of chromosomal fragments (40–42). The condensin II complex is closely linked to this mechanism, not only due to the fact that the capacity of regulating chromosome cohesion and segregation during mitosis, but also owing to that recently being shown to be intimately involved in molecular mechanisms closely related to tumor progression, such as histone regulation (43). Considering that, as mentioned above, uncontrolled proliferative capacity represents the fundamental behavior of malignant tumors and based on the consensus that tumors are essentially a genetic disease (44). NCAPG2, a subunit of the chromosomal condensin II complex, is increasingly recognized for its role in tumor evolution and might act as a pan-oncogene with a unique role in the evolution of various cancers.

We confirmed this possibility based on a pan-cancer expression analysis of the TCGA and GTEx databases, with NCAPG2 being statistically significantly and aberrantly highly expressed in almost all cancers except KIRP, KICH, THCA. Furthermore, we performed COX regression analysis and KM survival curves confirmed, to a certain extent, that NCAPG2 might possess the potential to serve as a reliable biomarker. When further compared with the results of survival analysis, we found that NCAPG2 was significantly associated with four prognostic indicators, OS, DSS, DFS and PFS, in four different tumors, including LIHC, PAAD, KIRP and KIPAN, which highlights the emphasis on further research into the internal mechanisms. For instance, Meng et al. had explored the effect strategy of NCAPG2 in LIHC, and found that NCAPG2 not only stimulates STAT3 phosphorylation, but also potentiates NF-κB signaling, forming positive feedback with STAT3/NF-κB signaling pathway, stimulating the malignant progression of LIHC (16). Besides, the role of NCAPG2 in the context of LGG was also explored in depth by Wu et al, who concluded that NCAPG2 regulates the phosphorylation level of HbO1, as a known oncogene, and activates Wnt/β-catenin signaling, promoting malignant proliferation of LGG cells (17). The biomarker potential of NCAPG2 is also reflected in the fact that its expression varies between the different pathological stages of the same tumor, with higher expression in the advanced stage of the tumor, which equally supported by the ROC diagnostic curve based on NCAPG2 expression levels and thus in a pan-cancer perspective.

An established consensus in the perception of tumors is that neoplastic tissue is likely to act as an organ of greater complexity than otherwise healthy tissue, building up its own microenvironment to boost its varied properties (38, 45). Immune cells and stromal cells constitute the mainstay of TME with significant roles in a broad range of aggressive malignant progressions including the proliferation and invasion of tumors and drug resistance (45). A better deciphering of the biological code underlying TME could facilitate better and more precise tumor immunotherapy, which is considered to be a practical and powerful weapon against many refractory malignancies. Notably, prior studies by Jiang et al. focused on this and pointed to NCAPG2 as a potential target for immune infiltration therapy through a correlation analysis approach, which was similarly confirmed by the study by Ren et al. on glioma, which reinforced the prospective role of NCAPG2 in the field of immunotherapy (18, 46). Neither study, nevertheless, has done more to analyze other immune features, such as immunomodulatory genes. To this end, our study focuses on the relationship between NCAPG2 and the immune microenvironment, building on the analysis described above, thus assessing the role of NCAPG2 in immune cell infiltration and its relevance to TME. For that reason, we first assessed this by three immune scores, including StromalScore, ImmuneScore and EstimateScore. In pan-cancer, NCAPG2 correlated strongly with these three scores. Overall, however, the correlation was mostly negative, for example in GBM, where it was the highest correlation among the pan-cancers, which may explain, to some extent, the crucial role of NCAPG2 in GBM, as mentioned above. Future insights into such mechanisms might offer an effective immunotherapeutic option for GBM patients. Identical results have been observed in LUSC, which is consistent with existing studies of NCAPG2 in the context of LUSC. So far as the present conclusions are concerned, the differences in the correlation between the three separate scores might depict a conceptual map formed by immune and stromal cells in various malignancies, thus explaining the differential role of NCAPG2 in the prognosis of patients with diverse tumors. Targeting immune checkpoint genes like PD-1 and PD-L1, immune checkpoint blockade is currently among the mainstream immunotherapies (47, 48). The present study found that NCAPG2 displayed a significant correlation with ICI-related genes in most cancers, with a predominantly positive correlation. For instance, CD274 was positively associated with NCAPG2 in multiple tumors such as LGG, BLCA and BRCA. We infer that NCAPG2, as a pan-oncogene, may be able to enhance its malignancy by forming positive feedback with certain immune checkpoint genes, enabling tumor cells to be immune surveillance free. Significantly, the upregulation of ICI-related genes would be accompanied by a diminished anti-tumor effect of T cells. Taken together, these mechanisms might make targeting NCAPG2 for precision medicine a possibility in the future.

Not only that, but we further analyzed the correlation between NCAPG2 and the infiltration of a broad range of immune cells. It is straightforward to conclude that there is a significant correlation between NCAPG2 expression levels and numerous immune cells, such as T cells, B cells, neutrophils, macrophages, etc. Although the causality of such a connection in different malignancies could not be determined at this time, it lays the groundwork for further probing the mechanisms involved. An interesting and striking observation here is that CD8+ T cells displayed a strong positive correlation with THYM in all three distinct algorithms. What is known is that the infiltration of relatively high CD8+ T cells into TME represents a favorable prognostic feature in numerous malignancies (49). Together with the survival analysis mentioned above, namely that NCAPG2 is a protective factor in THYM patients. One is justified to infer that there exists an influential mechanism between NCAPG2 and CD8+ T cells that influences the improvement of survival prognosis of THYM patients awaiting further exploration. In addition, the correlation between NCAPG2 and immunomodulatory genes, including MHC, chemokine, and chemokine receptor related genes, similarly confirmed its closeness to tumor immunity, which was strengthened by phenomenon that apparent elevation of MHC-1 expression could be observed when NCAPG2 expression was downregulated. This confirms the established suspicion that downregulation of MHC-I is frequently a means of survival for cancer patients to resist immunotherapy from the inside out, a tactic that is thought to be one of the critical mechanisms by which tumor cells currently evade immune surveillance (50–52). In PDAC, MHC-I tends to be reduced due to the lysosome-dependent autophagic pathway (53). The above combined explain the current findings of the WB trial, which likely deals with the malignant attenuation of PC cells. However, the current results are very general, as our study did not go further to assess the changes in each MHC-related gene, nor was it able to explain the detailed causes of such changes. Even if these results could be interpreted reasonably, it remains to be seen whether they could be of as much translational medical value as possible (54).

Whether TMB, MSI or tumor purity, all are thought to influence the efficacy of treatment with immune checkpoint therapy (55, 56). A correlation between NCAPG2 and the three ICI therapeutic markers mentioned above was analyzed in our study, which shows significant correlations between NCAPG2 expression in STAD, GBM, LUAD, KICH and ACC tumors, suggesting the potential for further tumor classification based on these three markers or for specific therapeutic treatment. In part, to some degree, this hints at the need for further large-scale clinical trials to verify the impact of NCAPG2 expression levels on the prognosis of immunotherapy in patients. Additionally, we assessed the relationship between the stemness index and NCAPG2 and found that it was significantly correlated with NCAPG2 in pan-cancer, which exhibited parallels with prognostic outcomes, suggesting that NCAPG2 influences stemness progression. Known as the silent assassin, very few effective treatments are available for pancreatic cancer. Hence, taking PC as an entry point, we analyzed that NCAPG2 could be an independent prognostic criterion for PC patients. Moreover, we predicted the possible specific mechanisms through the pathway, and further explored the possibility that knockdown of the NCAPG2 gene could significantly reduce the proliferation, migration and invasion capacity of tumor cells, confirming that this effect of NCAPG2 on the malignant proliferation of tumor cells is realized to some degree through the impact on the EMT process. The above multi-omics combined analysis implied that NCAPG2 might pose a great potential as a novel prognostic and immune infiltration marker or therapeutic target for carcinomas, whereas the current studies are limited by the lack of in-depth experiments to verify its specific immune infiltration relationship with the TME, and the insufficiency of verification of the relevance of the current immunotherapeutic targets. Although the current study has, to a certain extent, demystified NCAPG2 in the field of immunotherapy from a macroscopic perspective, the current findings are even more suggestive of the necessity for further experimental work.

To sum up, NCAPG2, as a pan-oncogene, differentially expressed in different malignancies, could reflect the survival prognosis of patients with diverse tumors and act as a therapeutic and prognostic marker for a variety of malignancies. Also, NCAPG2 exhibited remarkable correlation with both immune cell infiltration and immune checkpoint genes, hinting that the potential as a target for tumor immunotherapy.

## Data availability statement

The original contributions presented in the study are included in the article/**Supplementary Material**. Further inquiries can be directed to the corresponding author.

## Author contributions

QW designed the manuscript, QW, ZZL, SZ, ZRL, and XH prepared the figure and drafted this manuscript; QW and XH performed the experiments; SZ, ZRL, YH, YZ, XZ, and YT enhanced the figures and language; MX edited and revised manuscript. All authors contributed to the article and approved the submitted version.

## References

[1] Rahib L Wehner MR Matrisian LM Nead KT. Estimated projection of US cancer incidence and death to 2040. JAMA Netw Open (2021) 4(4):e214708. doi: 10.1001/jamanetworkopen.2021.4708 33825840 PMC8027914

[2] Siegel RL Miller KD Fuchs HE Jemal A. Cancer statistics, 2022. CA Cancer J Clin (2022) 72(1):7–33. doi: 10.3322/caac.21708 35020204

[3] Fitzgerald RC Antoniou AC Fruk L Rosenfeld N. The future of early cancer detection. Nat Med (2022) 28(4):666–77. doi: 10.1038/s41591-022-01746-x 35440720

[4] Liu XS Yang JW Zeng J Chen XQ Gao Y Kui XY. SLC2A1 is a diagnostic biomarker involved in immune infiltration of colorectal cancer and associated with m6A modification and ceRNA. Front Cell Dev Biol (2022) 10:853596. doi: 10.3389/fcell.2022.853596 35399515 PMC8987357

[5] Zou Y Xie J Zheng S Liu W Tang Y Tian W. Leveraging diverse cell-death patterns to predict the prognosis and drug sensitivity of triple-negative breast cancer patients after surgery. Int J Surg (2022) 106936. doi: 10.1016/j.ijsu.2022.106936 36341760

[6] Hanahan D. Hallmarks of cancer: New dimensions. Cancer Discovery (2022) 12(1):31–46. doi: 10.1158/2159-8290.CD-21-1059 35022204

[7] Balon K Sheriff A Jacków J Łaczmański Ł. Targeting cancer with CRISPR/Cas9-based therapy. Int J Mol Sci (2022) 23(1):573. doi: 10.3390/ijms23010573 PMC8745084 35008996

[8] Stine ZE Schug ZT Salvino JM Dang CV. Targeting cancer metabolism in the era of precision oncology. Nat Rev Drug Discovery (2022) 21(2):141–62. doi: 10.1038/s41573-021-00339-6 PMC8641543 34862480

[9] Kagami Y Yoshida K. The functional role for condensin in the regulation of chromosomal organization during the cell cycle. Cell Mol Life Sci (2016) 73(24):4591–8. doi: 10.1007/s00018-016-2305-z PMC11108269 27402120

[10] Yuen KC Gerton JL. Taking cohesin and condensin in context. PloS Genet (2018) 14(1):e1007118. doi: 10.1371/journal.pgen.1007118 29370184 PMC5784890

[11] Kim JH Shim J Ji MJ Jung Y Bong SM Jang YJ. The condensin component NCAPG2 regulates microtubule-kinetochore attachment through recruitment of polo-like kinase 1 to kinetochores. Nat Commun (2014) 5:4588. doi: 10.1038/ncomms5588 25109385

[12] Zhan P Xi GM Zhang B Wu Y Liu HB Liu YF. NCAPG2 promotes tumour proliferation by regulating G2/M phase and associates with poor prognosis in lung adenocarcinoma. J Cell Mol Med (2017) 21(4):665–76. doi: 10.1111/jcmm.13010 PMC5345611 27862966

[13] Liu W Tanasa B Tyurina OV Zhou TY Gassmann R Liu WT. PHF8 mediates histone H4 lysine 20 demethylation events involved in cell cycle progression. Nature. (2010) 466(7305):508–12. doi: 10.1038/nature09272 PMC3059551 20622854

[14] Shiheido H Naito Y Kimura H Genma H Takashima H Tokunaga M. An anilinoquinazoline derivative inhibits tumor growth through interaction with hCAP-G2, a subunit of condensin II. PloS One (2012) 7(9):e44889. doi: 10.1371/journal.pone.0044889 23028663 PMC3441599

[15] Feng Z Zhang L Liu Y Zhang W. NCAPG2 contributes to progression of malignant melanoma through regulating proliferation and metastasis. Biochem Cell Biol (2022) 100(6):437–84. doi: 10.1139/bcb-2022-0048 36265182

[16] Meng F Zhang S Song R Liu Y Wang J Liang Y. NCAPG2 overexpression promotes hepatocellular carcinoma proliferation and metastasis through activating the STAT3 and NF-κB/miR-188-3p pathways. EBioMedicine. (2019) 44:237–49. doi: 10.1016/j.ebiom.2019.05.053 PMC6606561 31176678

[17] Wu J Li L Jiang G Zhan H Zhu X Yang W. NCAPG2 facilitates glioblastoma cells' malignancy and xenograft tumor growth *via* HBO1 activation by phosphorylation. Cell Tissue Res (2021) 383(2):693–706. doi: 10.1007/s00441-020-03281-y 32897418

[18] Jiang S Huang J He H Liu Y Liang L Sun X. NCAPG2 maintains cancer stemness and promotes erlotinib resistance in lung adenocarcinoma. Cancers (Basel) (2022) 14(18). doi: 10.3390/cancers14184395 PMC9497119 36139554

[19] Saidak Z Soudet S Lottin M Salle V Sevestre MA Clatot F. A pan-cancer analysis of the human tumor coagulome and its link to the tumor immune microenvironment. Cancer Immunol Immunother. (2021) 70(4):923–33. doi: 10.1007/s00262-020-02739-w PMC10991611 33057845

[20] Chen H Li C Peng X Zhou Z Weinstein JN Liang H. A pan-cancer analysis of enhancer expression in nearly 9000 patient samples. Cell. (2018) 173(2):386–99.e12. doi: 10.1016/j.cell.2018.03.027 29625054 PMC5890960

[21] Xie J Zhang J Tian W Zou Y Tang Y Zheng S. The pan-cancer multi-omics landscape of FOXO family relevant to clinical outcome and drug resistance. Int J Mol Sci (2022) 23(24):15647. doi: 10.3390/ijms232415647 36555288 PMC9778770

[22] Liu XS Kui XY Gao Y Chen XQ Zeng J Liu XY. Comprehensive analysis of YTHDF1 immune infiltrates and ceRNA in human esophageal carcinoma. Front Genet (2022) 13:835265. doi: 10.3389/fgene.2022.835265 35401696 PMC8983832

[23] Shen W Song Z Zhong X Huang M Shen D Gao P. Sangerbox: A comprehensive, interaction-friendly clinical bioinformatics analysis platform. iMeta. (2022) 1(3):e36. doi: 10.1002/imt2.36 PMC10989974 38868713

[24] Wei L Jin Z Yang S Xu Y Zhu Y Ji Y. TCGA-assembler 2: software pipeline for retrieval and processing of TCGA/CPTAC data. Bioinformatics. (2018) 34(9):1615–7. doi: 10.1093/bioinformatics/btx812 PMC5925773 29272348

[25] The ICGC/TCGA Pan-CancerAnalysis of Whole Genomes Consortium. Pan-cancer analysis of whole genomes. Nature (2020) 578(7793):82–93. doi: 10.1038/s41586-020-1969-6 32025007 PMC7025898

[26] Weinstein JN Collisson EA Mills GB Shaw KR Ozenberger BA Ellrott K. The cancer genome atlas pan-cancer analysis project. Nat Genet (2013) 45(10):1113–20. doi: 10.1038/ng.2764 PMC3919969 24071849

[27] Chandrashekar DS Bashel B Balasubramanya SAH Creighton CJ Ponce-Rodriguez I Chakravarthi B. UALCAN: A portal for facilitating tumor subgroup gene expression and survival analyses. Neoplasia. (2017) 19(8):649–58. doi: 10.1016/j.neo.2017.05.002 PMC5516091 28732212

[28] Thorsson V Gibbs DL Brown SD Wolf D Bortone DS Ou Yang TH. The immune landscape of cancer. Immunity. (2018) 48(4):812–30.e14. doi: 10.1016/j.immuni.2018.03.023 29628290 PMC5982584

[29] Yoshihara K Shahmoradgoli M Martínez E Vegesna R Kim H Torres-Garcia W. Inferring tumour purity and stromal and immune cell admixture from expression data. Nat Commun (2013) 4:2612. doi: 10.1038/ncomms3612 24113773 PMC3826632

[30] Li T Fu J Zeng Z Cohen D Li J Chen Q. TIMER2.0 for analysis of tumor-infiltrating immune cells. Nucleic Acids Res (2020) 48(W1):W509–w14. doi: 10.1093/nar/gkaa407 PMC7319575 32442275

[31] Zeng D Ye Z Shen R Yu G Wu J Xiong Y. IOBR: Multi-omics immuno-oncology biological research to decode tumor microenvironment and signatures. Front Immunol (2021) 12:687975. doi: 10.3389/fimmu.2021.687975 34276676 PMC8283787

[32] Racle J de Jonge K Baumgaertner P Speiser DE Gfeller D. Simultaneous enumeration of cancer and immune cell types from bulk tumor gene expression data. Elife. (2017) 6:e26476. doi: 10.7554/eLife.26476 PMC5718706 29130882

[33] Finotello F Mayer C Plattner C Laschober G Rieder D Hackl H. Molecular and pharmacological modulators of the tumor immune contexture revealed by deconvolution of RNA-seq data. Genome Med (2019) 11(1):34. doi: 10.1186/s13073-019-0638-6 31126321 PMC6534875

[34] Ballhausen A Przybilla MJ Jendrusch M Haupt S Pfaffendorf E Seidler F. The shared frameshift mutation landscape of microsatellite-unstable cancers suggests immunoediting during tumor evolution. Nat Commun (2020) 11(1):4740. doi: 10.1038/s41467-020-18514-5 32958755 PMC7506541

[35] Georgiadis A Durham JN Keefer LA Bartlett BR Zielonka M Murphy D. Noninvasive detection of microsatellite instability and high tumor mutation burden in cancer patients treated with PD-1 blockade. Clin Cancer Res (2019) 25(23):7024–34. doi: 10.1158/1078-0432.CCR-19-1372 PMC6892397 31506389

[36] Beroukhim R Mermel CH Porter D Wei G Raychaudhuri S Donovan J. The landscape of somatic copy-number alteration across human cancers. Nature. (2010) 463(7283):899–905. doi: 10.1038/nature08822 20164920 PMC2826709

[37] Malta TM Sokolov A Gentles AJ Burzykowski T Poisson L Weinstein JN. Machine learning identifies stemness features associated with oncogenic dedifferentiation. Cell. (2018) 173(2):338–54.e15. doi: 10.1016/j.cell.2018.03.034 29625051 PMC5902191

[38] Hanahan D Weinberg RA. Hallmarks of cancer: the next generation. Cell. (2011) 144(5):646–74. doi: 10.1016/j.cell.2011.02.013 21376230

[39] Yang L Li N Xue Z Liu LR Li J Huang X. Synergistic therapeutic effect of combined PDGFR and SGK1 inhibition in metastasis-initiating cells of breast cancer. Cell Death Differ (2020) 27(7):2066–80. doi: 10.1038/s41418-019-0485-4 PMC7308369 31969692

[40] Artandi SE DePinho RA. Telomeres and telomerase in cancer. Carcinogenesis. (2010) 31(1):9–18. doi: 10.1093/carcin/bgp268 19887512 PMC3003493

[41] Lim CJ Cech TR. Shaping human telomeres: From shelterin and CST complexes to telomeric chromatin organization. Nat Rev Mol Cell Biol (2021) 22(4):283–98. doi: 10.1038/s41580-021-00328-y PMC8221230 33564154

[42] Akincilar SC Chan CHT Ng QF Fidan K Tergaonkar V. Non-canonical roles of canonical telomere binding proteins in cancers. Cell Mol Life Sci (2021) 78(9):4235–57. doi: 10.1007/s00018-021-03783-0 PMC8164586 33599797

[43] Kim E Gonzalez AM Pradhan B van der Torre J Dekker C. Condensin-driven loop extrusion on supercoiled DNA. Nat Struct Mol Biol (2022) 29(7):719–27. doi: 10.1038/s41594-022-00802-x 35835864

[44] Nam AS Chaligne R Landau DA. Integrating genetic and non-genetic determinants of cancer evolution by single-cell multi-omics. Nat Rev Genet (2021) 22(1):3–18. doi: 10.1038/s41576-020-0265-5 32807900 PMC8450921

[45] Xie J Zheng S Zou Y Tang Y Tian W Wong CW. Turning up a new pattern: Identification of cancer-associated fibroblast-related clusters in TNBC. Front Immunol (2022) 13:1022147. doi: 10.3389/fimmu.2022.1022147 36275659 PMC9583405

[46] Ren W Yang S Chen X Guo J Zhao H Yang R. NCAPG2 is a novel prognostic biomarker and promotes cancer stem cell maintenance in low-grade glioma. Front Oncol (2022) 12:918606. doi: 10.3389/fonc.2022.918606 35898895 PMC9309203

[47] Morad G Helmink BA Sharma P Wargo JA. Hallmarks of response, resistance, and toxicity to immune checkpoint blockade. Cell. (2021) 184(21):5309–37. doi: 10.1016/j.cell.2021.09.020 PMC8767569 34624224

[48] Liu XS Liu JM Chen YJ Li FY Wu RM Tan F. Comprehensive analysis of hexokinase 2 immune infiltrates and m6A related genes in human esophageal carcinoma. Front Cell Dev Biol (2021) 9:715883. doi: 10.3389/fcell.2021.715883 34708035 PMC8544599

[49] Philip M Schietinger A. CD8(+) T cell differentiation and dysfunction in cancer. Nat Rev Immunol (2022) 22(4):209–23. doi: 10.1038/s41577-021-00574-3 PMC9792152 34253904

[50] Dhatchinamoorthy K Colbert JD Rock KL. Cancer immune evasion through loss of MHC class I antigen presentation. Front Immunol (2021) 12:636568. doi: 10.3389/fimmu.2021.636568 33767702 PMC7986854

[51] Taylor BC Balko JM. Mechanisms of MHC-I downregulation and role in immunotherapy response. Front Immunol (2022) 13:844866. doi: 10.3389/fimmu.2022.844866 35296095 PMC8920040

[52] Dersh D Hollý J Yewdell JW. A few good peptides: MHC class I-based cancer immunosurveillance and immunoevasion. Nat Rev Immunol (2021) 21(2):116–28. doi: 10.1038/s41577-020-0390-6 32820267

[53] Yamamoto K Venida A Yano J Biancur DE Kakiuchi M Gupta S. Autophagy promotes immune evasion of pancreatic cancer by degrading MHC-I. Nature. (2020) 581(7806):100–5. doi: 10.1038/s41586-020-2229-5 PMC7296553 32376951

[54] Cornel AM Mimpen IL Nierkens S. MHC class I downregulation in cancer: Underlying mechanisms and potential targets for cancer immunotherapy. Cancers (Basel). (2020) 12(7):1760. doi: 10.3390/cancers12071760 PMC7409324 32630675

[55] Addeo A Friedlaender A Banna GL Weiss GJ. TMB or not TMB as a biomarker: That is the question. Crit Rev Oncol Hematol (2021) 163:103374. doi: 10.1016/j.critrevonc.2021.103374 34087341

[56] Rizzo A Ricci AD Brandi G. PD-L1, TMB, MSI, and other predictors of response to immune checkpoint inhibitors in biliary tract cancer. Cancers (Basel) (2021) 13(3):558. doi: 10.3390/cancers13030558 PMC7867133 33535621

