# Supplementary figures and images for: NCAPG2 could be an immunological and prognostic biomarker: From pan-cancer analysis to pancreatic cancer validation
Source: Front Immunol. 2023 Jan 27;14:1097403. doi: 10.3389/fimmu.2023.1097403 (PMC9911455; doi:10.3389/fimmu.2023.1097403)

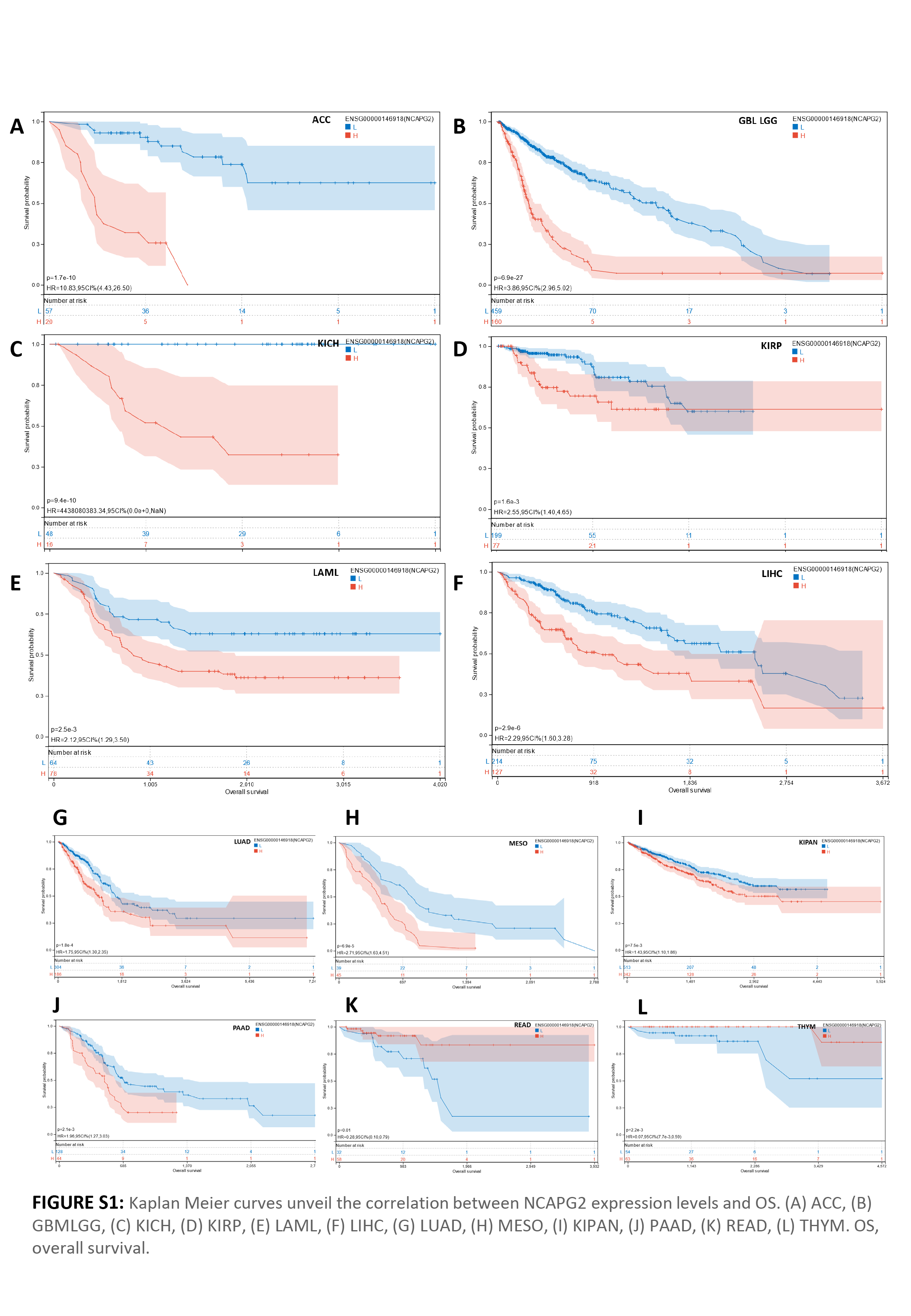

Supplement: Supplementary file 1 [file Image_1.tif]

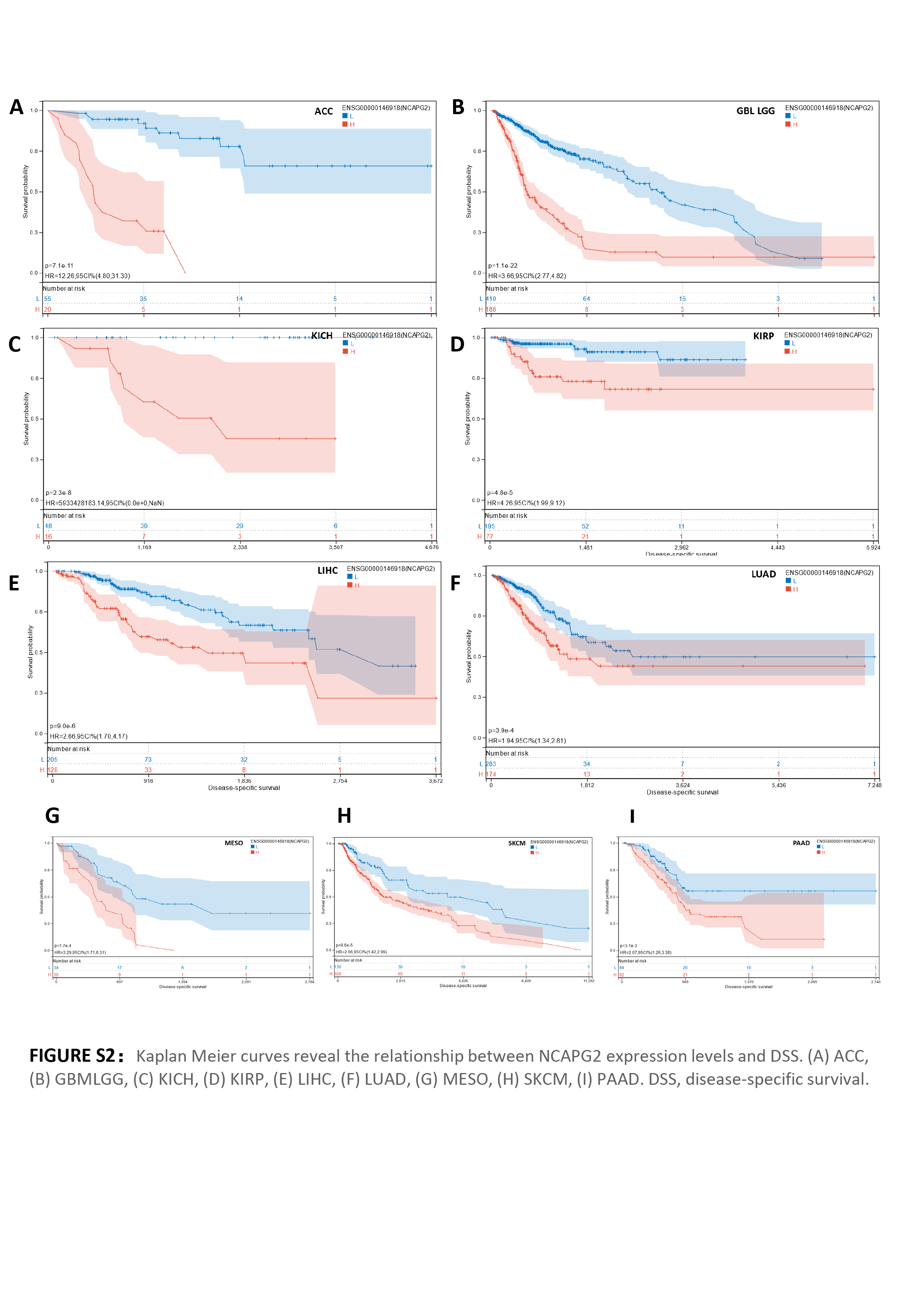

Supplement: Supplementary file 2 [file Image_2.tif]

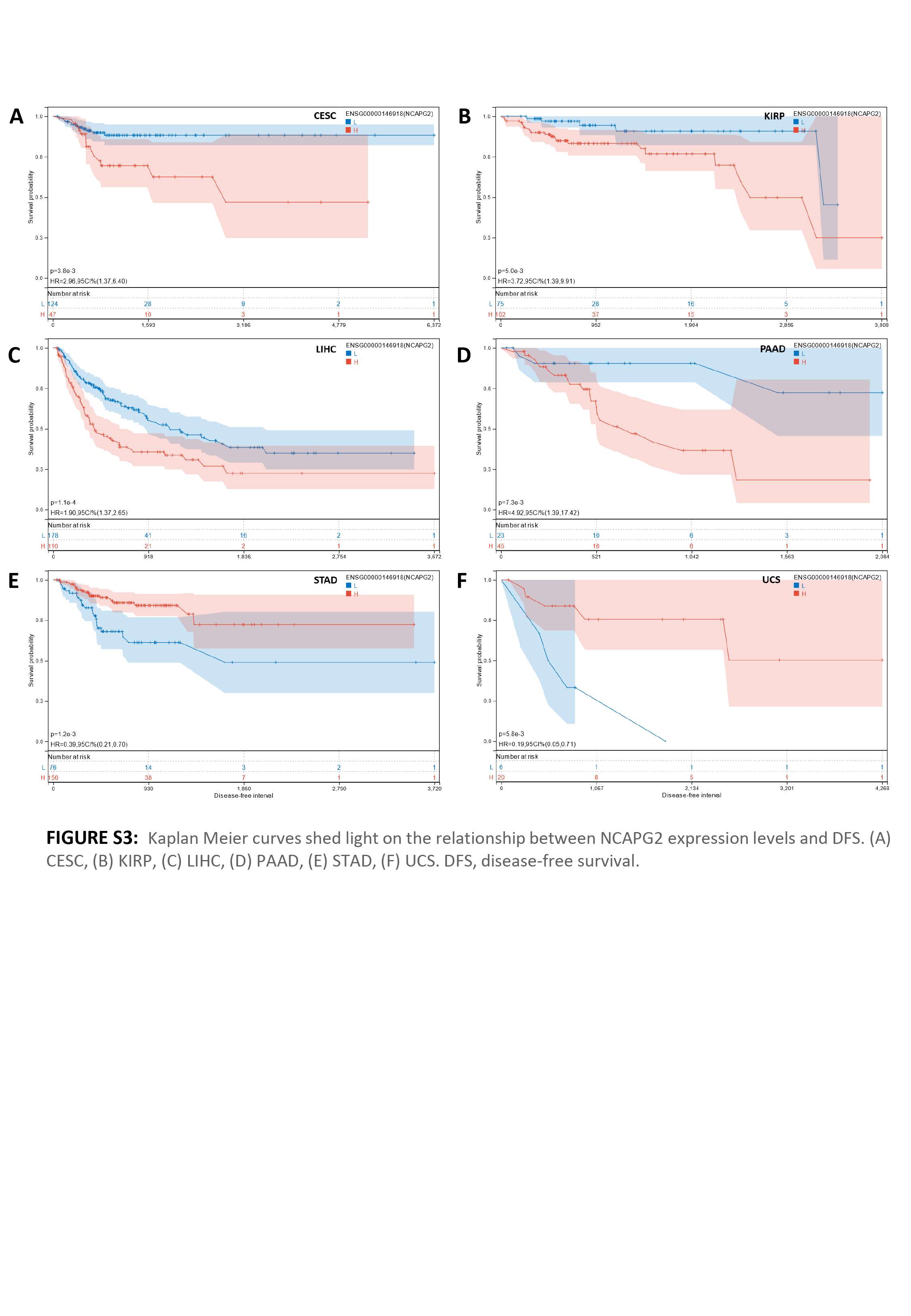

Supplement: Supplementary file 3 [file Image_3.tif]

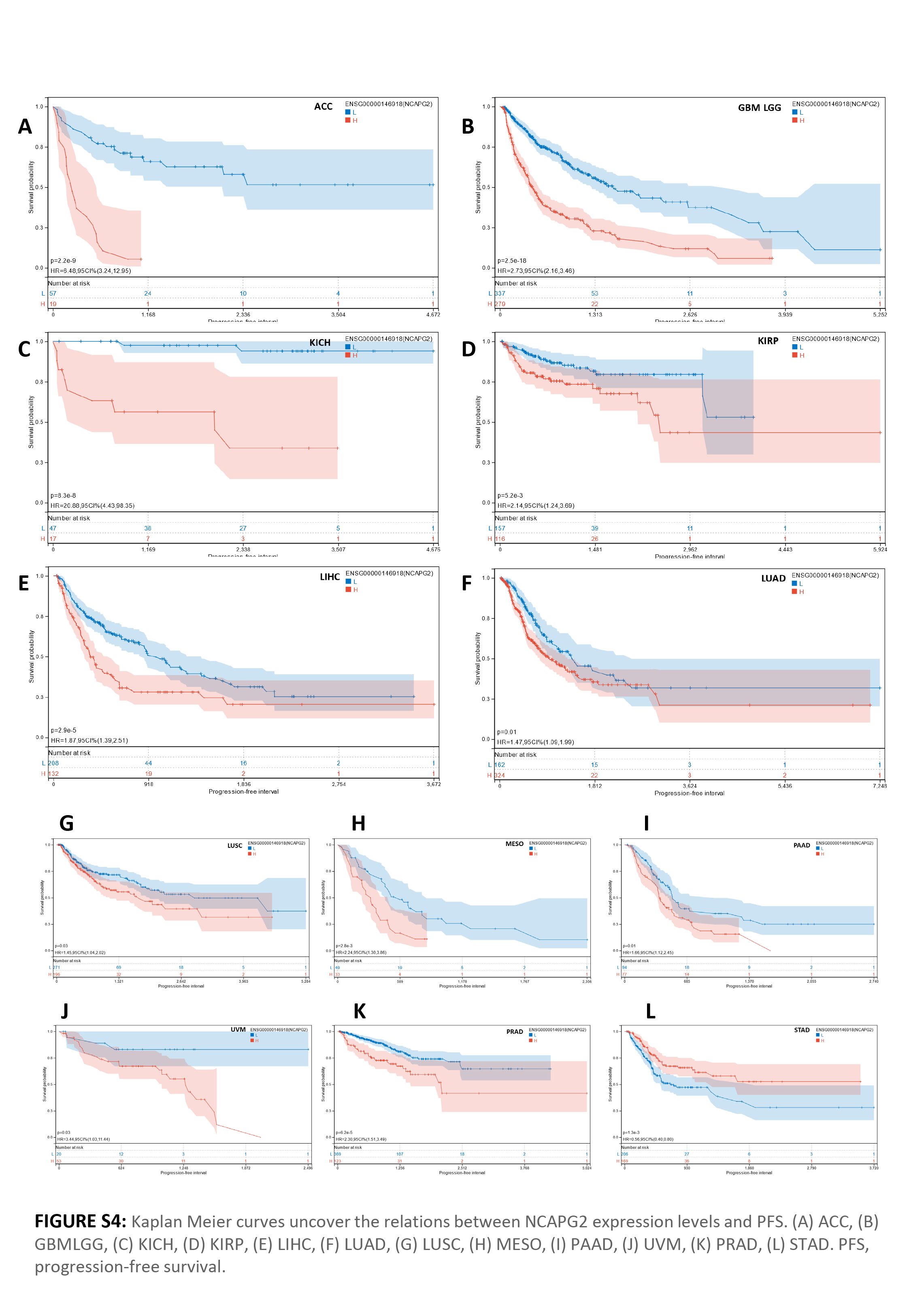

Supplement: Supplementary file 4 [file Image_4.tif]

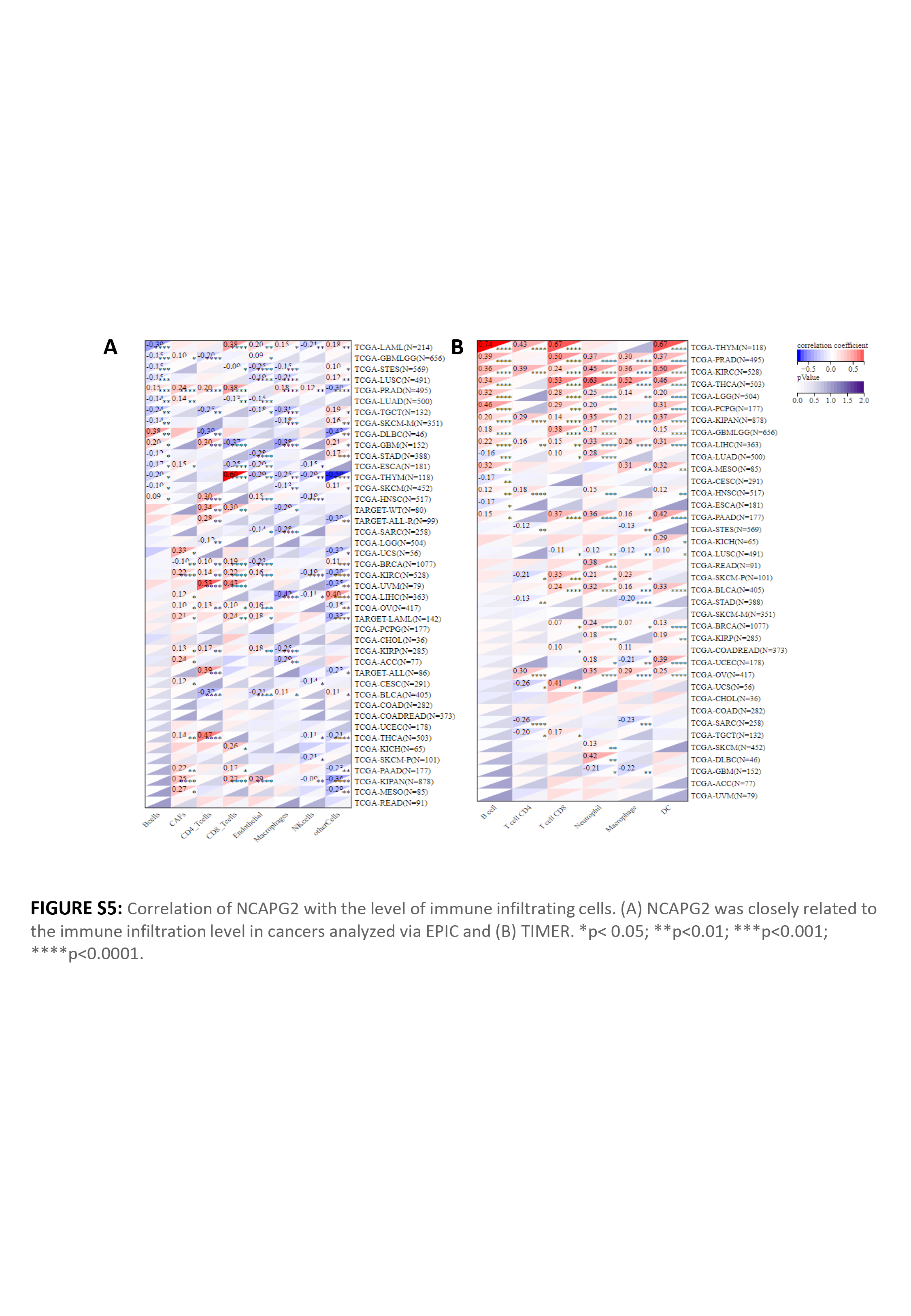

Supplement: Supplementary file 5 [file Image_5.tif]
